# Bullying in the clinical setting: Lived experiences of nursing students in the Central Region of Ghana

**DOI:** 10.1371/journal.pone.0257620

**Published:** 2021-09-23

**Authors:** Sara Ama Amoo, Awube Menlah, Isabella Garti, Evans Osei Appiah

**Affiliations:** 1 Cape Coast Teaching Hospital, Cape Coast, Ghana; 2 Valley View University, Accra, Adenta, Ghana; Sunway University, MALAYSIA

## Abstract

**Introduction:**

Nursing students are confronted with bullies in the classroom and during clinical placement. Acquisition of the necessary psychomotor skills intended during clinical placements may be impeded when workplace bullies intimidate students. This study aimed to describe the various bullying behaviours experienced by nursing students and their effects during clinical placement in the Central Region of Ghana.

**Methods:**

A qualitative phenomenological descriptive approach using a semi-structured interview guide was employed to collect data from nursing students in focus groups. Overall, six (6) focus groups were used, with five (5) students in each group comprising males and females. The sample size was based on data saturation and was saturated on the six focus group discussions giving a sample size of 30. Purposive sampling was used to select students who had been on the ward at least three clinical placements and had experienced bullying in the clinical setting. In-depth interviews were conducted, recorded, transcribed verbatim and analysed using content analysis.

**Results:**

The study revealed that nursing students had experienced bullying practices such as shouting, isolation, humiliation and being assigned tasks below their competency level. In addition, findings showed that bullying led to a loss of confidence and caused stress and anxiety in nursing students.

**Conclusion:**

Therefore, it is recommended that nursing students are mentored holistically in a caring and accepting environment where they will be supported to achieve their learning goals, build their confidence, and develop their personal and professional identity.

## Introduction

Bullying in the health care system has existed for ages and is still being practised in most healthcare facilities [1,2]. Apart from fighting microbes on a daily basis, most nurses are engaged in a never-ending battle with colleagues [1–4]. Nurses face several obstacles when discharging their duties, including bullying and hostility [5,6]. Studies indicate a rising prevalence in nurse to nurse hostility [3–8]. Intra professional violence is a challenge for a profession such as nursing that embodies caring; unfortunately, bullying is more common in nursing than in any other health profession [1,9,10]. It has been documented that a nurse can be a potential bully, tolerate, support or reinforce bullying [11–13]. Interestingly, nursing students are not spared as they are exposed to bullying both in the classroom and during clinical placements [5,7]. Existing studies show that nursing students are easy targets for bullies due to the subordinate nature of nursing, unfamiliar work environments, underreporting of bullying behaviour and limited clinical experience [l3,17]. Disturbingly, some students enter the nursing profession expecting to be bullied and thus are on their guard, ready to fight back and defend themselves from being bullied [14]. These students can inherit the bullying culture and transmit it when they attain positions of power, resulting in a repetitive vicious cycle [14]_._ Hence it is recommended that nurses treat their colleagues well to help end this behaviour [15].

Bullying is the process of subjecting victims to several negative behaviours [16]. Bullying is aggressive or harmful acts or behaviours carried out repeatedly over time and directed at someone who finds it difficult to defend him or herself [13]. Bullying can be in the form of physical or psychological violence, which is usually the modus operandi, and it has been ascertained to negatively affect the outcome of health care [17,18]. Most adult workplace bullies resort to psychological violence as it may go unnoticed by the authorities for sanctioning [17,19].

The woes of nursing students begin immediately they step foot into the clinical area [4,5,7]. A recent study has reported that 50% of nursing students were bullied during clinical placement [8]. For instance, it has been reported that some nursing students are verbally abused and face other forms of bullying during their clinical practice [19,20]. What is worrying is that bullying tends to get worse over time. Another study in Canada further revealed that third and fourth-year nursing students experienced bullying more than first and second year students [11,21]. This was attributed to the fact that third and fourth-year students had spent more time in the clinical setting than the first and second-year students. The perpetrators who victimise the students are usually in positions of power; thus, they thrive because all reports and grievances often pass through their hands. Any attempt by the student to voice out will only be greeted with worsening encounters leading to psychological distress among the victims [22,23].

Throughout the literature, there are several bullying behaviours identified [24–28]. However, the following were noted as behaviours targeted more often to students, including nursing students, which could result in absenteeism and loss of interest in the nursing profession. It is reported that verbal abuse is what bullies often use to keep students in check [10,14,20]. Nevertheless, in nursing, these acts are considered inappropriate as they affect the psychological well-being of nursing students. These include but are not limited to being yelled at, belittled, spoken to rudely, gossip, ridiculed, persistent criticism, blaming, being made the subject of crude jokes, being assigned duties that fall below or above one’s professional capabilities and false allegations[16,29–31].

Some nursing students have sustained direct bodily harm on the ward [23]. Others described their clinical placement experience as their worst nightmare because they were left to chart a course of their own with little supervision from their preceptors [21]. While some purported bullies may not lash out intentionally at nursing students, others think they are quite justified because they perceive such acts as providing an avenue for new nurses to show their worth and earn respect [32,33]. Bullying, however, does not entail offering diverse opinions, giving guidance, educating staff, constructive criticism, performing managerial duties and ensuring safety at the workplace [12].

In general, the rippling effect of bullying is numerous and transcends professional and personal boundaries, with the patient at the centre feeling helpless and powerless [1]. On average, more time is spent at the workplace than in any other social setting. Workplace environments should be cordial enough to enhance work and increase productivity. Bullying can create a toxic work environment that is very volatile [30,31]. Issues such as decreased job satisfaction, absenteeism, tardiness, lack of teamwork, increased errors, poor communication and collaboration are detrimental effects on the health institution associated with workplace bullying [18,31]. Globally, there is an increasing trend of nursing shortage, especially during this pandemic. Evidence shows nurses who are bullied leave the profession to other fields, further worsening shortages and stretching health systems beyond capacity [4]. The concomitant stress, anxiety and low self-esteem from bullying lead to maladaptation in social relationships inside and outside the work environment [7–9]. Additionally, nurses who are bullied exhibit uncaring attitudes to their patients and become less compassionate, thus compromising quality care [34,35]. The bystander is not left untouched as nurses who witness others being bullied may look on helplessly and become frustrated [36].

Literature lucidly points to the negative impact it tends to have on students. Bullying can take a toll on an individual’s physical and psychological well-being. Whilst some cry, keep quiet, withdraw from patients, become invisible, depressed and nonchalant, others also develop a hatred for the profession and may look for a means of branching out [8,28]. Victims who had been bullied experienced insomnia, low self-esteem, and overuse of the sick leave as an escape route from work [32,37,38]. Although not commonly reported in the literature, suicidal tendencies can germinate from bullying [24].

Some qualitative studies have unveiled the bullying experiences of some nursing students [39–41]. Some of these experiences include hostility by senior staff, being ridiculed by senior staff and workplace violence [42]. These behaviours have been ascertained to expose victims to various forms of physical and psychological harm that may affect their work output and patient’s outcome [43].

In most Sub-Saharan African countries like Ghana, children are socialised not to challenge authority [44]. These norms have made their way into nursing classrooms. The first nurse trainees in colonial Ghana were trained by British nurses who permeated a senior-junior relationship [45]. Juniors had very little freedom, limited job performance roles and were expected to obey before complaining [2]. Today submissiveness is still an integral part of nursing in Ghana. Student nurses on clinical placement are expected to be submissive to the older experienced nurses [7,8]. As such, when students raise issues of being bullied, they are labelled as being disrespectful and not serious. They are expected to adapt to bullying behaviours, especially from senior colleagues. These days there are mixed reactions amongst the new generation of Ghanaian nurses concerning bullying. Whilst some feel that bullying is wrong and must not be permitted on any grounds, others urge students to accept such behaviours as a normal part of their clinical experience which will stop once they become fully qualified nurses [46–49].

Recently, a study in Ghana ascertained the presence of bullying among nurses in Ghana. Relatives of patients were found to be involved in bullying against Ghanaian nurses [50,51]. Again, a study in Ghana discovered that 52.2% of their participants experienced verbal abuse [51]. The study further revealed that some of the bullying were sexual harassment by medical doctors (12%). In addition, some nursing students suggested that they are only allowed to check vital signs irrespective of their level during their clinical attachment [52]. However, literature regarding bullying among nursing students in Ghana is scarce; hence, the researchers intended to explore the lived experiences of nursing students in the Central Region of Ghana regarding bullying.

## Methodology

### Study design

To understand the phenomenon of bullying, a qualitative phenomenological descriptive design using focus groups was employed [53]. This approach and the method is appropriate if one wishes to understand subjective, "lived" experiences, and gain insights into people’s action and reaction to a phenomenon [48]. This method and design assisted the researchers to explore participants experiences regarding bullying in the clinical setting. This helped provide a detailed understanding of bullying among nurses and revealed the effect of workplace bullying among nursing students. Findings are expected to inform staff nurses to treat their junior colleagues with care, employers to supervise and address bullying issues appropriately, and appropriate institutions to formulate policies regarding bullying in the nursing profession to help decrease the effects associated with bullying among nursing students in the clinical setting. A purposive sampling technique was used to recruit participants to help researchers select participants who have had clinical experience and reported having experienced a form of bullying.

### Participants and setting

Generic nursing students pursuing general nursing programmes were selected from two nursing educational institutions in the Central Region of Ghana, namely the University of Cape Coast School of Nursing and the Cape Coast Nursing and Midwifery Training College (NMTC). Both schools offer general nursing, and their students undertook their clinical placement at the Cape Coast Teaching Hospital and the Cape Coast Metropolitan Hospital. The Nursing and Midwifery Training Colleges have a three year programme leading to the award of a diploma, and students start clinical placement from the first year. On the other hand, the university has a four-year general nursing programme leading to the award of a degree, and students commence clinical placement in the second year. A purposive sampling technique was used to recruit 30 participants. The purposive sampling allowed researchers to recruit participants who met the inclusion criteria and were legible to provide appropriate responses for the study but not just due to their availability. The focus groups were formed by grouping students from the same class together, comprising of both males and females whilst adhering to the criteria for inclusion. The focus groups also helped researchers to retrieve complex experiences and varied responses regarding the phenomenon under study—the groups comprised of 5 students in a group. The focus group helped ensured uniformity of participants and allow for different perspectives regarding bullying. Data collection continued until saturation where no new data was retrieved from the groups. Data saturation was achieved on the sixth group giving a sample size of 30. The data was collected by the researchers who served as moderators. The data collection lasted for two months, and each interview lasted for 45–60 minutes.

### Criteria for inclusion

The following inclusion criteria were used: (a) second and third-year students from the Cape Coast Nursing and Midwifery Training College; (b) third and fourth-year students from the University of Cape Coast. Each participant had at least three clinical placements and had experienced bullying during their clinical attachment. Additionally, participants should be willing to partake in the study and give their consent.

### Ethical consideration

Ethical clearance was sought from the University of Cape Coast Institutional Review Boardand a protocol ID number (UCC IRB 30/5/2014) was given. Permission was also sought from the Cape Coast Nursing and Midwifery Training College (NMTC) before the commencement of the study.

### Data collection procedure

The researchers visited the selected schools on different days with the ethical clearance letter. Together with one of the school’s administrators, permission was sought from the tutors and lecturers to have access to the students after their scheduled classes. After a brief introduction regarding the study, students who had such experiences were to wait behind after their class hours. The researchers hanged around till the end of the class. The researchers first introduced themselves to the students and allowed them to introduce themselves to make them comfortable. After the introduction, they were informed about the purpose, inclusion criteria, data collection process and the significance of the study. Contact numbers of those eligible who were willing to partake in the study were taking and a time was rescheduled with them based on their availability on school premises when school has closed. Only participants who had been bullied during their clinical attachment were selected. Participants were taken through the full data collection process and were assured of voluntary withdrawal at any stage of the process if they felt that some issues were too sensitive to discuss without prejudice. The interview responses were treated with full confidentiality, and participants were urged not to discuss the matters raised in the interview session outside the group. Participants were also asked not to identify themselves during the interview session. Only participants in a focus group and researchers were available at the time of data collection.

The focus group discussions were audio-taped. Validation of the data collected was achieved by addressing credibility, confirmability, dependability and transferability. These were achieved by transcribing all recorded data verbatim, allowing only participants who met the inclusion criteria to be recruited into the study, ensuring a non-threatening environment for them to air their experiences and giving of pseudonyms to conceal their identities. Additionally, researchers served as only moderators, describing the setting, design, data collection procedure, sampling technique and sample size were utilised.

### Instrument/Tool for data collection/sample size

Study participants were put in focus groups, and data were collected using in-depth semi-structured interviews. A semi-structured interview guide was used to steer the interviews. The interview guide had eight (8) open-ended questions with probes. There were two sections; Section A helped retrieve information about participants socio-demographic background, and section B retrieved information about the experiences of bullying and bullying effects. There were six focus groups in the study and five students in each group, making the sample size 30, by which time saturation was reached because the responses were redundant. All participants completed the study. The interviews were carried out by the researchers. Participants were allowed to sign consent forms before the commencement of the interviews. To encourage narration, the participants were asked broad, open-ended questions. They were asked to talk about their experiences, both positive and negative, in a non-threatening manner. This set the tone for the participants to open up and generally speak about their workplace experiences before probing further into the kind of negative experiences they had come across. During the interview session, when a question was asked, participants were allowed time to respond. Participants were assigned pseudonyms by giving numbers in the group from 1–5 to conceal their identity. This was necessary because since the data was to be published, their personal information will be made known. Furthermore, no one could identify them even if they get access to the recorded data. Group members were also informed about the rules of the FGDs, which included; not sending information during the discussion outside after discussion, waiting for a member to finish making their point before coming in, and not arguing with other members. All the participants were given an opportunity to contribute, and follow up questions were asked to clarify statements made and also to probe for further responses and explanations. Data collection lasted for 45-60minutes per session.

### Data analysis

The authors’ tape-recorded participants responses with a tape recorder. The researchers analysed the data concurrently with data collection using content analysis. All researchers took part in the data analysis. The recorded data was shared among the researchers to transcribe verbatim. There were six FGDs, hence the first and second authors transcribed two of the transcripts each whilst the other two left were transcribed by the fourth and the fifth authors. The data was transcribed verbatim. Each researcher analysed the data that was transcribed by them, which all researchers later reviewed. The data transcribed was first read thoroughly by the researchers severally to familiarise themselves with the data. Clarifications were made where necessary by contacting appropriate participants to make sure the meanings of participants were maintained. After familiarisation, the researchers grouped the responses according to the study objectives. The researchers further read the data to assign meanings to the content. Similar meanings were then put together. Themes were formulated for similar meanings, and subthemes were placed under the main themes. COREQ (Consolidated Criteria for Reporting Qualitative Research) checklist guided the reporting of the findings.

## Results

### Demographic data

Thirty students were recruited for the study. Twenty of the participants were females, whilst ten were males. In both schools, females formed the majority of the student population; therefore, there was no deliberate attempt to sample more females than males. Five of the participants were aged 20 and below, and the remaining twenty-five were between the ages of 21 to 25 years. Two nursing institutions were used from the Central Region of Ghana, which was the University of Cape Coast (2nd-4th year nursing students) and Nursing and Midwifery Training College in Cape Coast (2nd to 3rd year Nursing Students).

### Themes

Two themes and 7 subthemes in all emerged from the study analysis. The themes are Bullying Behaviour experienced by nursing students during their clinical attachment and effects of Bullying. The following subthemes emerged from the bullying behaviour: shouting at the students, Neglect of student nurses by staff nurses, Humiliation and Assignment below competency level. The subthemes formulated under the Effects of bullying were: Loss of confidence, Stress and Effects on learning outcomes.

### Theme 1: Bullying behaviours

Four patterns of behaviour emerged when asked to describe the various bullying and bullying behaviours they experienced during clinical placement. Students mentioned shouting, isolation, humiliation, and being assigned work below their competency level as their commonly experienced bullying behaviours.

### Shouting at students

All the participants reported encountering bullying as they have experienced it at one time or another. In addition, the majority of the students shared that they have experienced shouting and described it as a bad experience that should be stopped.

*“The nurses on the wards shout at us all the time*. *When they want to ask you to do something they shout at you*, *when you try to take initiative they shout and ask you who told you to do that*?*” FGD1*, *P2*

*"Hmm with bullying*, *it is like maltreatment and I have experienced this severally by being shouted at by some nurses*, *some doctors and even some patient relatives" FGD 5*, *P1*.

*“Some of the nurses even though they know your name and you have the name tag on*, *they will still address you heeeeer*‥*student*, *when they are call you like that*, *it is so annoying*. *They sometimes they belittle you even in-front of patients and their relatives "FGD2*, *P5*.

Due to the pervasive nature of shouting on the wards, few students who had experienced it did not see it to be wrong with the perception that it puts some students in check. A student explained as follows:

*"I also agree that shouting at students is not good but sometimes we students deserve to be disciplined a little*, *I think that it keeps us on our toes*, *but maybe if they take us to the nurses’ room and correct us that would be better" FGD 3*, *P4*.

*“I think it is ok for them to shout sometimes*. *We are dealing with human lives and at that point that you are making a mistake or doing something wrong if they don’t shout to stop you*, *you could harm the patient but how*, *where and when to shout should be takeninto consideration*.*" FGD6*, *P1*

### Neglect of student nurses by staff nurses

Students in this study also reported that they were ignored during nursing duties and not allowed to partake in procedures performed, which is a form of bullying in the clinical setting. The majority of the participants were worried about being exempted from procedures and being ignored.

*“When we go to the wards*, *there are times we meet some nurses who will just ignore us*. *They will go about performing their procedures without saying a word to you; they neglect you as if you are not part of the ward and as if you do not exist*, *how will you feel if you were the one*?*” FGD1*, *P3*

*"What pains me is that sometimes when they are performing procedures*, *they will ask you to stay outside as if you are the patient relative*, *when you refuse to because you want to learn*, *they will say you are stubborn to the extent of even punishing you for no reason*. *FGD5*, *P5*

Other students shared their thoughts as follows;

*“Well*, *I think that we are ignored because of mistrust*, *they don’t seem to believe in us that we can perform the procedures correctly*. *Others also want things done quickly during their shift*, *and therefore they don’t like to involve students who will slow them down*. *But if they keep doing that how can we become perfect and improve upon our skills*?*”*. *FGD3*, *P5*

*“Some nurses have the perception that the university nursing students only know book or the theoretical aspect of nursing and do not pay attention to practicals and so they do not see the essence of helping us with the practicals*. *But if you don’t teach us how will we learn*? *Besides*, *we will take over from them the next too far future*.*” FGD 2*, *P2*

Few recounted that they were not able to defend themselves but just to permit themselves to be subjected to the bullying.

*"Sometimes I wish I could revenge but as a junior*, *who are you to defend yourself or talk back*? *You just have to keep quiet and endure*. *" FGD3*, *P19*

*"There was a time I reported and I thought the nurse who did that would be called*, *but nothing of such happened till we completed the clinical practise and left and since then I have never reported such act before" FGD4*, *P3*.

### Humiliation

Participants in this study recounted how they were humiliated and spoken to in a demeaning manner in the presence of patients and other nurses for making a mistake or not performing an assigned procedure right.

*“We students are learning to become nurses*, *we may be inexperienced and we may make mistakes but we certainly are not stupid but on the wards we are made to feel stupid sometimes*. *I was once told in the presence of other people you don’t know anything*, *you can’t even perform simple tasks*. *Are you sure you can be a nurse*?*”*. *FGD4*, *P4*

*“Some nurses will only allow student nurses to clean stool*, *urine and bath patients throughout the shift and will not teach you any other thing else*. *Sometimes when it happened like that*, *the patients and relatives do not respect you because they think you don’t know anything; that is why you do that always*.*”FGD 1*, *P5*

*“It is so disheartening that sometimes the senior nurses will only be sending you on errands*. *Once they know you are a student*, *sometimes they will not even address you properly when they are calling you to send you”FGD6*, *P5*.

Another student pointed out how some nurses just made fun of the students.

*“I went to the ward with one female colleague of mine to this hospital for a vacation clinical*. *My colleague had already worn her apron and cap; when we got to the ward and greeted*, *the nurse in-charge retorted*, *the way you have dressed in all white with your cap on*, *are you a chef*? *and she added that the students have come again*, *they don’t know anything just coming to be gallivanting” FGD4*, *P1*

### Assignment below competency level

According to some of the participants who indicated that they were allowed to perform some duties, they reported those duties were outside their objectives.

*“When you are assigned to do errands it means that the whole day you will carry patients to do investigations*, *go to pharmacy and you will not learn anything so by the time you return to school*, *you will not know anything about your objectives*, *the main reason why you went for the clinical*.*” FGD6*, *P2*

*“Sometimes when you go the ward*, *they willonly allow youto be folding gauze and mould cotton which is not a procedure while they will be chatting and sitting at the nurses’ station doing nothing”FGD5*, *P4*.

### Effects of bullying

In describing how bullying affected them, participants spoke about how bullying behaviours took a toll on them. Their responses centred on loss of confidence, stress and poor learning outcomes.

### Lack of confidence

People usually gain confidence and a sense of optimism when they are encouraged, and their little efforts are recognised. However, the participants in this study reported that not being acknowledged and appreciated by the nurses who were to serve as supervisors affected their confidence. According to the participants, the feeling of rejection or humiliation made them lose their self-confidence, which affected their ability to practice and emotional status.

*“We are often reminded that we are not competent*, *so we are not confident to do things independently*. *Because of that it kills our confidence to the extent that even when you are assigned a task and you are doing the right thing you are not sure of yourself and you will be hesitating*. *All these affect our confidence and it makes us timid”*. *FGD4*, *P5*

*“Because we are always maltreated*, *ignored and not guided to perform procedures as expected*, *sometimes when they are overwhelmed with activities and they ask you to assist them to perform some tasks on patients*, *sometimes you go there and you do not know what to do because you’re confused*.*”FGD3*, *P3*

*“As for me I was not taught anytime during my clinical practise session*, *so I do not know anything to be frank*. *Due to this*, *I have only worked on dummies but not patients*. *This could affect my confidence level when I am asked to take care of patients after completion*.*”FGD2*, *P3*

### Stress

Bullying has the potential of making students feel stressed. Working under stress can make these student nurses engage in acts of negligence and malpractices, which could be detrimental to the patients’ health outcomes. This can also expose the nurses to some legal issues. Besides, it may affect their ability to learn and process information during their clinical practice. In the following extracts, participants described how stressful bullying could be.

*“You don’t know what to expect*, *you wonder*: *will they be receptive*, *will they be kind to me or they will be mean to me*, *so whenever you are about to go for clinical practise*, *you will be worried*. *"FGD1*, *P5*.

*“You get fatigued before you close from work*, *because all tedious works like lifting they will call you*. *sometimes you even feel that you have chosen the wrong profession” FGD2*, *P2*

### Effects on learning and patient outcomes

Students nurses performance is significantly determined by the ability to learn theoretically and apply the knowledge they acquire in the clinical environment to improve their skills. Bullying student nurses can cause them to be truants, take unnecessary excuses and instil fear in them which could affect their learning. Participants pointed out that bullying in the clinical setting affected learning. The following statements indicate how students learning outcomes on the wards were being affected.

*“I feel that as a student we learn better when we get the opportunity to explore*, *but here is the case you are even scared to explore*.*Sometimes those procedures that we learn in school*, *we are supposed to try our hands on them at the hospital but rather they engage us in other things*, *so our skills are being curtailed”*. *FGD4*, *P2*

*“How can I even learn when I am afraid of them*. *I suggest they change their attitudes towards us to learn better” FGD5*, *P3*.

*“For me*, *I suggest for us to be able to learn well*, *they should be welcoming and caring so that we feel at home*, *they should learn to correct us the right way" FGD 6*, *P3*.

Some participants also narrated that bullying goes a long way to affect the care provided to patients.

*Most health workers are not discharging their duties well on the ward*, *which affect the health outcomes of patients because of the fear of the staff during their clinical practice*, *which hindered them from learning well and perfecting their skills FGD1*, *P5*.

*"How can you be in the right frame of mind to take care of your patients when you are personally disturbed because of intimidation from your superiors*?. *Sometimes you may even serve wrong medications or do something wrong*.*"*. *FGD2*, *P4*

*"Sometimes you are even tempted to divert your anger on the patients and their relatives when your own senior colleagues are maltreating you*, *and this attitude would mar the nurse-patient relationship and affect the recovery of patients"*. *FGD 5*, *P5*

## Discussion

Regarding the demographic characteristics, it was identified that females formed the majority of the participants, thus 20 (66.6%). This could be attributed to the fact that the number of males entering the nursing profession is lower than that of females in Ghana. This was consistent with a study that revealed that females formed the majority in the nursing profession [54]. Although females formed the majority of the study participants, most of the participants were young. It was noted that gender and age did not play a role in bullying experienced by students. Participant responses indicated that being a student was reason enough to be subjected to bullying. Bullying of nursing students is, however, not gender-specific, and it’s targeted at all students. This is congruent to findings from a study that reported that neither age nor sex played a role in students’ bullying experiences of students [14]. On the contrary, it was reported that age played a role in bullying and that students who were younger got bullied more than older students [11]. The current study results that age had no significant relation with bullying experiences was not expected since in the Ghanaian culture, people who are older are treated with respect and dignity whilst younger ones are often disrespected and maltreated.

The present study discovered several bullying experiences among the participants. The bullying experiences identified were; shouting, isolation, humiliation, and assigned work below their competency level. Shouting was unveiled as the most common bullying behaviour experienced by all students in the clinical setting. Similarly, shouting is mentioned as one of the most commonly occurring bullying behaviour in the clinical setting in several other studies [2,16,21]. This finding suggests that nursing students are not communicated appropriately by other nurses and probably due to stereotyping of their behaviour. This could be influenced by the Ghanaian culture where superiors are viewed as bosses, and juniors are to obey without complaining since doing so will be misinterpreted as disrespect [2]. Due to this, some even term bullying as a form of shouting as normal. However, in professional dialogue, shouting must not be encouraged in the least form since it may negatively affect nursing care and patient outcomes.

Another bullying behaviour identified was isolation, which students described as being excluded from procedures, nurses refusing to speak to students and refusing to teach or supervise students. Participants recounted missed learning opportunities and stagnation at competency levels because they were isolated and not given a chance to develop their skills. It was reported that the forms of isolation students experienced included being totally ignored, students’ efforts not being acknowledged, and students not being spoken to. This present study’s findings supported findings of other studies where nursing students were excluded from some activities on the ward and experienced other forms of bullying [55,56]. Excluding students from procedures or not being supervised or taught as identified in the current study could go a long way to affect the way these students will treat their junior colleagues since they will view it as a norm. Furthermore, students will feel reluctant to ask questions or even attend to patients who seek their assistance for fear of being shouted at or punished by their superiors, which may also affect patient care.

Other scholars have identified that isolation can lead to loss of concentration and impaired learning skills [46]. This was evident in the study as student nurses narrated not been involved in activities where skills acquisition was concerned. Participants also indicated that verbal communication was often demeaning. According to some of them, they were subjected to verbal humiliations by some senior nurses, which eroded their confidence. This finding is in consonance with findings of a study done in Turkey among nursing students where they faced verbal and physical abuse [57]. Some recounted being made to feel incompetent. Meanwhile, clinical placement is supposed to offer an opportunity for nursing students to build their confidence and develop their professional identity. This lack of confidence is likely to make students timid and affect their ability to take the initiative, think critically and ask questions that will enhance their job performance. It was suggested that nurses who have confidence in themselves tend to perform better at work [11]. Lack of confidence that emerged from the current study could affect the way these students would discharge their duties when employed to work in the various hospitals and may not want to take initiatives since they may be afraid of making mistakes or may likely engage in malpractices and negligence since they were not given opportunities by their superiors to perfect their skills and knowledge during their practice.

For students, clinical placement is an opportunity to learn new things and improve their nursing skills; however, they mentioned how they lost valuable time running errands because that is what they were seen to be good at. An assertion that one can have their professional status weakened or damaged when assigned worthless tasks; or made to perform procedures below their competence level; supports this statement [33,58]. In addition, some staff nurses are oblivious of the course content of the practicum placement. To curtail the habit of assigning students to work outside their scope of practice, nursing schools must send students to the wards accompanied by nursing faculty and clear objectives as to the desired learning outcomes. Sending students on errands during clinical practice could stem from the fact that the young are expected to be submissive and run errands for the elderly in the Ghanaian context; however, bringing this culture into nursing may negatively affect learning outcomes and patients care since nursing is teamwork.

With regards to the effects of bullying, stress and lack of confidence have been recognised as likely effects of bullying. According to the respondents, their negative experiences during their clinical practice lowered their confidence level and exposed them to unnecessary stress. This finding is congruent with other studies where stress, lack of enthusiasm, confidence, and reduced work performance was attributed to bullying [48,59]. This assertion was reiterated by participants of this study who detailed that they experienced both physical and psychological stress after being bullied, which affected their work output. This implies that more workplace errors and job dissatisfaction would be encountered, making clinical placement a highly stressful occurrence. In this era where the law holds health care workers liable in situations where there are negative outcomes, nursing students may not be spared, and errors may not go unpunished regardless of the contributing factors. Developing a caring and compassionate nature is part of the process of professional identity development. Students who experience a hostile work environment during their education are likely to become nurses who care less about their patients.

### Study limitations

The study was limited to only nursing students without considering the perpetrator’s experiences, making it a field of study for other researchers. The study also focused on only one Region in Ghana, making it a limitation since there are 16 regions in the country.

### Implications for behavioural health

In accordance with the International Council of Nurses guidelines, there should be respectful mentorship of nursing students. In view of the findings generated from this study, it is clear that changes must be made in clinical nursing education. In order for students to practice in their domain of pre-set objectives, the nursing faculty, the staff nurse, and the preceptor must work together. Preceptors must be trained, enumerated, and present in the clinical area to perform their guiding and supervisory roles. Again it is recommended that bullying be dealt with at an individual and institutional level. Individuals should be assertive and insist on their rights, and seek redress when they are bullied. Hospitals should have policies in place outlining what constitutes bullying and the consequences of bullying. Punitive measures should be put in place to discourage workplace bullying. This study concentrated on the bullying behaviours and bullying effects experienced by nursing students.

### Future research directives

Studies in other settings can examine the reasons for bullying and examine how those bullied copes with the situation. Again, bullying should be looked at from the point of view of those called bullies in this study: the ward in-charges and staff nurses. This will help determine why they employ bullying behaviours against students. In addition, this study did not examine the effect of bullying on patients. As bullying is common in the clinical area, it is important to consider the potential impact on patients at the centre of it all. Other studies can also look at Cyberbullying among nursing students.

## Conclusion

This study has unearthed important student perspectives about bullying in the clinical setting. Bullying behaviours shown to students during clinical placement are becoming incessant and detrimental. The damage to nursing students may be irreparable and result in a chain of repetitive behaviour whereby they become bullies in the not so distant future. Awareness of bullying behaviour would enable nursing students to recognise when they are being bullied so that appropriate solutions can be found. Nursing students deserve to be mentored in a caring and accepting environment where they would be supported to achieve their learning goals, build their confidence and develop their personal and professional identity. Bullying in nursing is unacceptable, and the profession should not tolerate bullying of any individual.

## Supporting information

S1 Checklist For qualitative studies COREQ checklist. (DOCX)

S1 File Interview guide. (DOCX)

## References

[1] Al-Ghabeesh SH, Qattom H. Workplace bullying and its preventive measures and productivity among emergency department nurses. BMC health services research. 2019 Dec;19(1):445. doi: 10.1186/s12913-019-4268-x 31269990 PMC6607587

[2] Akiwumi A, editor. Nursing education in Ghana for the 21st century. Woeli Pub. Services; 1994.

[3] Baltimore JJ. Nurse collegiality: fact or fiction?.Nursing management. 2006 May 1;37(5):28–36. doi: 10.1097/00006247-200605000-00008 16651900

[4] Bartholomew K. Ending nurse-to-nurse hostility: Why nurses eat their young and each other. HC Pro, Inc.; 2006.

[5] Blakey AG, Smith-Han K, Anderson L, et al. Interventions addressing student bullying in the clinical workplace: a narrative review. BMC medical education. 2019 Dec 1;19(1):220. doi: 10.1186/s12909-019-1578-y 31226986 PMC6588850

[6] Böhmig C. Ghanaian nurses at a crossroads: managing expectations on a medical ward 2010.

[7] Meierdierks-Bowllan N. Nursing students’ experience of bullying. Nurse Educ. 2015;40(4):194–8. doi: 10.1097/NNE.0000000000000146 25763780

[8] Budden LM, Birks M, Cant R, et al. Australian nursing students’ experience of bullying and/or harassment during clinical placement. Collegian. 2017 Apr 1;24(2):125–33. doi: 10.1016/j.nepr.2017.04.011 28456062

[9] Castronovo MA, Pullizzi A, Evans S. Nurse bullying: A review and a proposed solution. Nursing outlook. 2016 May 1;64(3):208–14. doi: 10.1016/j.outlook.2015.11.008 26732552

[10] Çelebioğlu A, Akpinar RB, Küçükoğlu S, et al. Violence experienced by Turkish nursing students in clinical settings: their emotions and behaviors. Nurse education today. 2010 Oct 1;30(7):687–91. doi: 10.1016/j.nedt.2010.01.006 20129722

[11] Clarke C. The effects of bullying behaviours on student nurses in the clinical setting. (2009)

[12] Dellasega C. When nurses hurt nurses: Recognising and overcoming the cycle of nurse bullying. Sigma Theta Tau; 2011.

[13] Cleary M, Hunt GE, Horsfall J. Identifying and addressing bullying in nursing. Issues in mental health nursing. 2010 Apr 1;31(5):331–5. doi: 10.3109/01612840903308531 20394479

[14] Curtis J, Bowen I, Reid A. You have no credibility: Nursing students’ experiences of horizontal violence. Nurse education in practice. 2007 May 1;7(3):156–63. doi: 10.1016/j.nepr.2006.06.002 17689439

[15] Daiski I. Changing nurses’ dis-empowering relationship patterns. Journal of Advanced Nursing. 2004 Oct;48(1):43–50. doi: 10.1111/j.1365-2648.2004.03167.x 15347409

[16] Einarsen S, Hoel H, Notelaers G. Measuring exposure to bullying and harassment at work: Validity, factor structure and psychometric properties of the Negative Acts Questionnaire-Revised. Work & stress. 2009 Jan 1;23(1):24–44.

[17] Etienne E. Exploring workplace bullying in nursing. Workplace Health & Safety. 2014 Jan;62(1):6–11. 24571049 10.1177/216507991406200102

[18] Farrell GA, Shafiei T. Workplace aggression, including bullying in nursing and midwifery: A descriptive survey (the SWAB study). International journal of nursing studies. 2012 Nov 1;49(11):1423–31. doi: 10.1016/j.ijnurstu.2012.06.007 22770947

[19] Farrell GA, Bobrowski C, Bobrowski P. Scoping workplace aggression in nursing: findings from an Australian study. Journal of advanced nursing. 2006 Sep;55(6):778–87. doi: 10.1111/j.1365-2648.2006.03956.x 16925626

[20] Kwon HJ, Kim HS, Choe KS, et al. A study on verbal abuse experienced at medical centers. Journal of Korean Clinical Nursing Research. 2007;13(2):113–24.

[21] Foster B, Mackie B, Barnett N. Bullying in the health sector: A study of bullying of nursing students. New Zealand Journal of Employment Relations. 2004;29(2):67.

[22] Home AN, Policy NI. Letter to the Editor by Kristian “Kiki” Divin, BSN, RN to “Psychological Distress and Workplace Bullying Among Registered Nurses”.

[23] Hopkins M, Fetherston CM, Morrison P. Prevalence and characteristics of aggression and violence experienced by Western Australian nursing students during clinical practice. Contemporary nurse. 2014 Dec 1;49(1):113–21. doi: 10.5172/conu.2014.49.113 25549752

[24] Garred P, Brygge K, Sorensen CH, et al. Mannan-binding protein—levels in plasma and upper-airways secretions and frequency of genotypes in children with recurrence of otitis media. Clinical & Experimental Immunology. 1993 Oct;94(1):99–104. doi: 10.1111/j.1365-2249.1993.tb05984.x 8403525 PMC1534361

[25] Iglesias M, Vallejo R. Prevalence of bullying at work and its association with self-esteem scores in a Spanish nurse sample. Contemporary nurse. 2012 Aug 1;42(1):2–10. doi: 10.5172/conu.2012.42.1.2 23050566

[26] Jackson D, Clare J, Mannix J. Who would want to be a nurse? Violence in the workplace–a factor in recruitment and retention. Journal of nursing management. 2002 Jan;10(1):13–20. doi: 10.1046/j.0966-0429.2001.00262.x 11906596

[27] Zapf D, Escartin J, Scheppa-Lahyani M, Einarsen SV, et al. Empirical findings on prevalence and risk groups of bullying in the workplace. Bullying and Harassment in the Workplace: Theory, Research and Practice. 2020 Apr 9:105.

[28] Johnson SL, Rea RE. Workplace bullying: concerns for nurse leaders. JONA: The Journal of Nursing Administration. 2009 Feb 1;39(2):84–90. doi: 10.1097/NNA.0b013e318195a5fc 19190425

[29] Iglesias M, Becerro de Bengoa Vallejo R. Prevalence of bullying at work and its association with self-esteem scores in a Spanish nurse sample. Contemporary nurse. 2012 Aug 1;42(1):2–10. doi: 10.5172/conu.2012.42.1.2 23050566

[30] Lewis M.A. Bullying in nursing.Nursing Standard, 2002. 15, 39–42. 10.7748/ns2001.07.15.45.39.c3064 12212387

[31] Lewis MA. Nurse bullying: organisational considerations in the maintenance and perpetration of health care bullying cultures. Journal of nursing Management. 2006 Jan;14(1):52–8. doi: 10.1111/j.1365-2934.2005.00535.x 16359446

[32] Longo J, Sherman RO. Leveling horizontal violence. Nursing Management. 2007 Mar 1;38(3):34–7. doi: 10.1097/01.numa.0000262925.77680.e0 17491129

[33] McKenna BG, Smith NA, Poole SJ, Coverdale JH. Horizontal violence: Experiences of registered nurses in their first year of practice. Journal of advanced nursing. 2003 Apr;42(1):90–6. doi: 10.1046/j.1365-2648.2003.02583.x 12641816

[34] Murray JS. No more nurse abuse. Let’s stop paying the emotional, physical, and financial costs of workplace abuse. American Nurse Today. 2008;3(7):17–9.

[35] Roche M, Diers D, Duffield C, Catling-Paull C. Violence toward nurses, the work environment, and patient outcomes. Journal of Nursing Scholarship. 2010 Mar;42(1):13–22. doi: 10.1111/j.1547-5069.2009.01321.x 20487182

[36] Simons S. Workplace bullying experienced by Massachusetts registered nurses and the relationship to intention to leave the organisation.Advances in Nursing Science. 2008 31, E48–E49. doi: 10.1097/01.ANS.0000319571.37373.d7 18497581

[37] Stevens S. Nursing workforce retention: Challenging a bullying culture. Health Affairs. 2002,21(5), 189–193. doi: 10.1377/hlthaff.21.5.189 12224882

[38] Stevenson K., Randle J., & Grayling I. Inter-group conflict in health care: UK students’ experiences of bullying and the need for organisational solutions. The Online Journal of Issues in Nursin. 2006,11(2), 18. doi: 10.3912/OJIN.Vol11No02Man0 17201580

[39] Allari RS, Farag MK. Nursing students’ expectations regarding clinical training: a qualitative study. Int J Nurs Sci. 2017;7(3):63–70.

[40] Zhu Z, Xing W, Lizarondo L, Guo M, Hu Y. Nursing students’ experiences with faculty incivility in the clinical education context: a qualitative systematic review and meta-synthesis. BMJ open. 2019 Feb 1;9(2):e024383. doi: 10.1136/bmjopen-2018-024383 30826795 PMC6398780

[41] Smith CR, Gillespie GL, Brown KC, Grubb PL. Seeing students squirm: nursing students’ experiences of bullying behaviors during clinical rotations. Journal of Nursing Education. 2016 Sep 1;55(9):505–13. doi: 10.3928/01484834-20160816-04 27560118 PMC5149060

[42] Simons SR, Mawn B. Bullying in the workplace—A qualitative study of newly licensed registered nurses. AAOHN journal. 2010 Jul;58(7):305–11. doi: 10.3928/08910162-20100616-02 20608570

[43] Gaffney DA, DeMarco RF, Hofmeyer A, Vessey JA, Budin WC. Making things right: Nurses’ experiences with workplace bullying—A grounded theory. Nursing research and practice. 2012 Jan 1;2012. doi: 10.1155/2012/243210 22567223 PMC3337490

[44] Khalil D. Levels of violence among nurses in Cape Town public hospitals. Nursing Forum. 2009, 44 (3), 207–217. doi: 10.1111/j.1744-6198.2009.00144.x 19691657

[45] Kisseih D. A. Developments in nursing in Ghana. International Journal of Nursing Studies. 1968, 5(3), 205–219. doi: 10.1016/0020-7489(68)90038-2 5187504

[46] Thobaben M. Horizontal workplace violence.Home Healthcare Management and Practice. 2007, 20(1), 82–83. doi: 10.1177/1084822307305723

[47] Townsend T. Breaking the bullying cycle. American Nurse today. 2012, 7(1); 12–15.

[48] Yildirim A., &Yildirim D. Mobbing in the workplace by peers and managers: Mobbing experienced by nurses working in health care facilities in Turkey and its effects on nurses. Journal of Clinical Nursing. 200716 (8), 1444–1453. doi: 10.1111/j.1365-2702.2006.01814.x 17655532

[49] Yildirim D. Bullying among nurses and its effects.International Nursing Review, 56, 504–511. doi: 10.1111/j.1466-7657.2009.00745.x 19930081

[50] Boafo IM, Hancock P. Workplace violence against nurses: a cross-sectional descriptive study of Ghanaian nurses. Sage open. 2017 Mar;7(1):2158244017701187.

[51] Boafo IM, Hancock P, Gringart E. Sources, incidence and effects of non-physical workplace violence against nurses in Ghana. Nursing open. 2016 Apr;3(2):99–109. doi: 10.1002/nop2.43 27708820 PMC5047339

[52] Adjei CA, Sarpong C, Attafuah PA, Amertil NP, Akosah YA. “We’ll check vital signs only till we finish the school”: experiences of student nurses regarding intra-semester clinical placement in Ghana. BMC nursing. 2018 Dec;17(1):1–6. doi: 10.1186/s12912-018-0292-0 29853798 PMC5975683

[53] Holloway I. & Wheeler S. Qualitative Research in Nursing and Healthcare(3rded.). 2010 Oxford, Wiley-Blackwell.

[54] Arif S, Khokhar S. A historical glance: Challenges for male nurses. JPMA The Journal of the Pakistan Medical Association. 2017 Dec 1;67(12):1889–94. 29256536

[55] Austin AM. A Phenomenological Examination of Perception of Incivility in Vocational Nurse Educators (Doctoral dissertation, Northcentral University).

[56] Ahn YH, Choi J. Incivility experiences in clinical practicum education among nursing students. Nurse education today. 2019 Feb 1;73:48–53. doi: 10.1016/j.nedt.2018.11.015 30504075

[57] Palaz S. Turkish nursing students’ perceptions and experiences of bullying behavior in nursing education. Journal of Nursing Education and Practice. 2013 Jan 1;3(1):23.

[58] Minton C, Birks M. “You can’t escape it”: Bullying experiences of New Zealand nursing students on clinical placement. Nurse education today. 2019 Jun 1;77:12–7. doi: 10.1016/j.nedt.2019.03.002 30913471

[59] Saidi LA, Othman MH. Socratic Dialogue Technique to Reduce the Level of Depression on Bully Victim in Workplace. Asian Journal of Behavioural Sciences. 2020 Mar 15;2(1):20–32.

