# Supplementary material for: Bullying in the clinical setting: Lived experiences of nursing students in the Central Region of Ghana
Source: PLoS One. 2021 Sep 23;16(9):e0257620. doi: 10.1371/journal.pone.0257620 (PMC8460007; doi:10.1371/journal.pone.0257620)
Supplement: S1 File — (DOCX) [file pone.0257620.s002.docx]

**Interview Guide**

***Dear Respondent,***

We are lecturers in some Public and Private Universities in the country and we are carrying out a research titled ‘**BULLYING IN THE CLINICAL SETTING: LIVED EXPERIENCES OF NURSING STUDENTS IN THE CENTRAL REGION OF GHANA,** In respect to this, we humbly implore your help in conducting this interview. The information will be tape recorded and your feedback will be kept strictly confidential and anonymous. Partaking in this study will not be harmful to your health. Safe precautions have been put in place to eradicate any potential risk. However, you hold the right to retreat from the exercise any time along the study. The result of the study will be of academic and public health benefit.

Thank you.

Demographic characteristics

1. Tell me all about yourself.

**Probes:**

 Age

 Marital status

 Number of children

 Educational level

 Employment status

 Ethnicity

**Section B: Bullying experiences**

2. What do you know about bullying in nursing?

3. What are some of the bullying behaviours experienced by you during your clinical attachment?

**Probes**

a. What do your superiors at the clinical setting do during your clinical placements or say that made you feel bad?

b. Why do you think such behaviours are meted out to you?

c. What are your views regarding the bullying behaviours?

d. Is it just you or other students are also treated poorly during clinical placement?

4. Are bullying behaviours easy to be reported in the clinical setting?

5. What were your reactions when you experience the bullying?

**Effects of bullying on learning and patients outcome**

6. How do these negative behaviours affect you or make you feel?

7. Are you able to complete your clinical placement under such circumstances? What changes do you make if any to be able to complete your clinical?

8. What are the long terms effects of bullying among students during their clinical practice?

Probes

a. How does this behaviour affect learning

b. How does this behaviour affect patients care delivery

c. How does this behaviour affect the interest and retention in nursing
